# RNAWRE: a resource of writers, readers and erasers of RNA modifications

**DOI:** 10.1093/database/baaa049

**Published:** 2020-07-01

**Authors:** Fulei Nie, Pengmian Feng, Xiaoming Song, Meng Wu, Qiang Tang, Wei Chen

**Affiliations:** 1 School of Life Sciences, Center for Genomics and Computational Biology, North China University of Science and Technology, 21 Bohai Road, Caofeidian Xincheng, Tangshan 063009, China; 2 School of Basic Medical Sciences, 1166 Liutai Avenue, Wenjiang District, Chengdu University of Traditional Chinese Medicine, Chengdu 611137, China; 3 Innovative Institute of Chinese Medicine and Pharmacy, Chengdu University of Traditional Chinese Medicine, 1166 Liutai Avenue, Wenjiang District, Chengdu 611137, China

## Abstract

RNA modifications are involved in various kinds of cellular biological processes. Accumulated evidences have demonstrated that the functions of RNA modifications are determined by the effectors that can catalyze, recognize and remove RNA modifications. They are called ‘writers’, ‘readers’ and ‘erasers’. The identification of RNA modification effectors will be helpful for understanding the regulatory mechanisms and biological functions of RNA modifications. In this work, we developed a database called RNAWRE that specially deposits RNA modification effectors. The current version of RNAWRE stored 2045 manually curated writers, readers and erasers for the six major kinds of RNA modifications, namely Cap, m^1^A, m^6^A, m^5^C, ψ and Poly A. The main modules of RNAWRE not only allow browsing and downloading the RNA modification effectors but also support the BLAST search of the potential RNA modification effectors in other species. We hope that RNAWRE will be helpful for the researches on RNA modifications.

Database URL: http://rnawre.bio2db.com

## Introduction

Since the first kind of RNA modification was discovered over 60 years ago (1), approximately 160 kinds of modifications present in RNA have been known at present (2). Without changing the sequence contents, RNA modifications not only participate in a series of biological processes, such as RNA localization and degradation (3), RNA splicing (4) and circadian rhythm (5), but also associated with several kinds of human diseases including metabolic diseases (6), cancer (7), cardiovascular diseases (8), etc. Despite advances having been made in our knowledge about RNA modifications, their biological functions are still incompletely understood.

The advent of next-generation sequencing technology facilitated the transcriptome-wide detection of several major kinds of RNA modifications (9, 10). In addition, these sequencing methods also enabled the discovery of RNA modification effectors that can catalyze, recognize and clear RNA modifications, including RNA modification deposition effectors (‘writers’), RNA modification recognition effectors (‘readers’) and RNA modification removal effectors (‘erasers’).

Besides dynamically installing, removing and interpreting RNA modifications, these effectors are also found to be linked with neurological diseases, metabolic diseases and mitochondrial-related defects (11). However, our understanding of these ‘writers’, ‘readers’ and ‘erasers’ is only at the beginning. Accordingly, the identification of RNA modification effectors will be helpful for understanding regulatory mechanisms and biological functions of RNA modification.

Although a huge amount of researches has been focused on RNA modifications (12–15), to the best of our knowledge, there is not a database systematically collecting the information of RNA modification effectors. Therefore, it is urgent to develop such a database, where the researchers could obtain the comprehensive information about the readers, writers and erasers of RNA modification.

**Figure 1:**
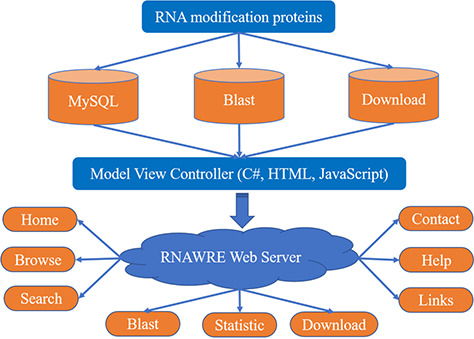
The schematic flow for the construction of RNAWRE.

Stimulated by a recent work (16), in the present study, we developed a database, called RNAWRE, that deposits the ‘writers’, ‘readers’ and ‘erasers’ for RNA modifications, which is available at http://rnawre.bio2db.com/. The current version of RNAWRE contains more than 2045 manually curated RNA modification enzymes from animals for the six major kinds of RNA modifications, namely Cap, m^1^A, m^6^A, m^5^C, ψ and Poly A. The construction of RNAWRE is illustrated in Figure 1.

**Table 1:** The summarizing information of RNAWRE

Modification	Readers (596)	Writers (1085)	Erasers (364)
	CBP20 (1)	RNMT(93)	DCP2(89)
Cap(185)	CBP80 (1)		DCP1 (1)
		TRMT61A(99)	
m^1^A(352)	-	TRMT61B(82)	ALKBH3(90)
		NSUN3(81)	
	YTHDC1(94)		
	YTHDF1(92)		
	YTHDF2(92)	METTL14(95)	
m^6^A(1057)	YTHDF3(92)	WTAP(93)	FTO(94)
	YTHDC2(86)	METTL3(91)	ALKBH5(90)
	HNRNPC (86)		
	HNRNPA2B1(52)		
m^5^C(95)	NA	NSun2(95)	NA
		PUS7(96)	
ψ(283)	NA	PUS3(95)	NA
		PUS1(92)	
Poly A(73)	NA	CFI(73)	NA

The number of effectors is indicated in the bracket.

The ‘NA’ represents the effectors that are not collected in RNAWRE.

## Database design and structure

### Data collection

To establish RNAWRE, according to the extracted RNA modification effectors from the literature, we firstly collected the seed sequences of the effectors for the six major kinds of RNA modifications, namely Cap, m^1^A, m^6^A, m^5^C, ψ and Poly A from GenBank. To identify the potential effectors for the other species, we also collected the protein sequences of animals from Ensembl (17). Based on these sequences, we performed BLAST by using the BLAST program (blast-2.7.1+) (18). And then, we matched the seed sequences to the downloaded animals’ proteins with the BLASTP program with an E-value cut-off of 10^−5^, sequence identity of 85% and the score of 200. By doing so, we obtained 2682 pieces of proteins might participate in RNA modification regulations. In order to obtain a high-quality dataset, we manually verified these proteins from the UniProt (19) database. Finally, we obtained 2045 proteins that are writers, readers and erasers of RNA modifications.

### Web interface implementation

The RNAWRE website was implemented by performing a variety of common software packages in the WINDOWS system, including Internet Information Services, MySQL database management, ASP.NET, HTML, CSS and JavaScript (20). The data were processed and analyzed by the C#, HTML and JavaScript. Highcharts (https://www.highcharts.com.cn/) were used for the statistic page. All proteins were stored in a MySQL database. An interactive web interface was constructed to enable users to conveniently access the RNAWRE and obtain the information needed either for basic research applications or biological analysis through any modern browser on their devices. C#, HTML and JavaScript were implemented to transmit user query information and rapidly extracted data from MySQL databases management to generate report pages.

**Figure 2:**
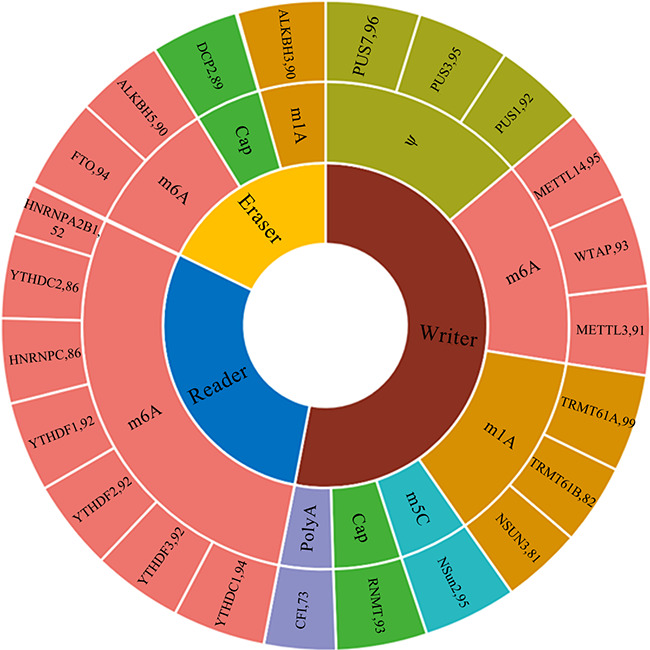
Statistical analysis of RNAWRE. The number effectors in each kind of modifications.

## Result and guidance of RNAWRE

The RNAWRE consists of nine main modules, i.e. Home, Browse, Search, Blast, Statistic, Download, Links, Help and Contact.

### Data statistics

At present, the RNAWRE database contains 2045 manually curated writers, readers and erasers for the six major kinds of RNA modifications, Cap, m^1^A, m^6^A, m^5^C, ψ and Poly A. The detail number of effectors related to each kind of modifications are 1057, 352, 283, 185, 95 and 73, respectively. The detail information was enumerated in Table 1. Among these proteins, 1085 are writers, 596 are readers and 364 are erasers, respectively. The sunburst chart (Figure 2) shows the number of effectors for the six major kinds of RNA modifications in RNAWRE. In this sunburst chart, the innermost ring is divided into three parts, representing the writers, readers and erasers of the RNA modifications, respectively. The outermost ring in the sunburst represents the RNA modification effectors, and the size of the fan represents the number of these effectors. The ring in the middle of the sunburst represents the RNA modification associated with the effectors.

**Figure 3:**
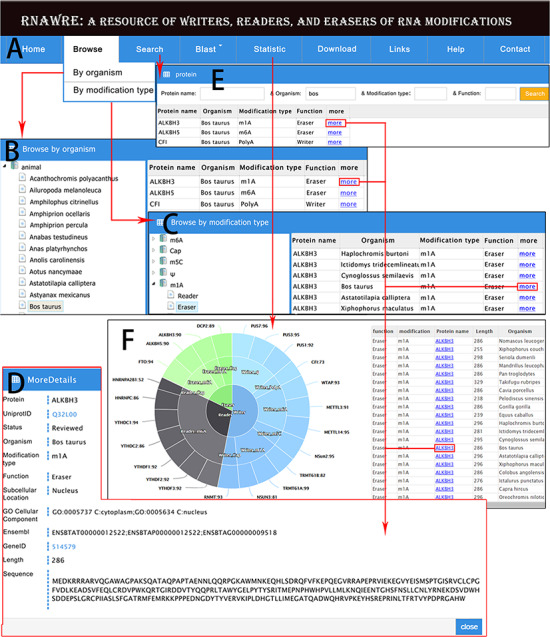
Web interface and usage of RNAWRE. (**A**) Home page. (**B**) Browse page by organism. (**C**) Browse page by modification type. (**D**) The detail page of RNA modification protein. (**E**) The search page of RNAWRE. (**F**) The statistical page.

### Browse

In the browse module embedded in the title bar (Figure 3A), users can browse RNA modification effectors by organism or modification type (Figure 3B and C). In the option of ‘Browse by organism’, the left of the page is the tree represents the Ensembl (17) taxonomy categories of animals. By clicking the organism such as ‘*Bos taurus*’, the RNA modification protein is displayed in the list on the right. By clicking on the ‘more’ button in the list, the detailed information of these proteins, such as UniProtID link to the UniProt database, review status, subcellular location if any, GO cellular component if any, Ensembl ID, GeneID if any link to the NCBI Gene database, length and sequence will be displayed in a new page (Figure 3D). In the option of ‘Browse by modification type’, on the left of the page is the tree represents the modification type and the function. By clicking the button, the RNA modification protein will be displayed in the list on the right, and clicking the more button can display the detail page (Figure 3C and D).

### Search

In the search module, RNAWRE allows the users to search proteins by ‘protein name’, ‘organism’, ‘modification type’ or ‘function’. Users can directly obtain the RNA modification proteins via input protein name or organism or modification type or function. Fuzzy match was masterly applied to cover all query possibilities. The filter can greatly help users obtain entries that meet their needs among the massive proteins (Figure 3D and E).

### Blast

The BLASTP tool was embedded in the RNAWRE database by using the BLAST-2.7.1+ program to help users perform sequence alignment (18). We provide a user-friendly graphic interface based on web forms. The BLAST database contained the whole RNA modification effectors that we collected. Amino acid sequences in FASTA format can be directly submitted by copying the data to the frame or uploading a FASTA file. Some parameters, such as the E-value, can be modified or simply set to the default parameter values by the users according to the research aims before performing a BLASTP search. Finally, after clicking the ‘Blastp’ icon, the results will be shown in a new page. Users can browse or download the BLAST results.

### Download and links

In the Download module, users can download the data deposited in RNAWRE. To facilitate scientific researchers in the relevant fields, in the Links module, we provided a resource page of other relevant researches about RNA modifications, such as the database Modomics, RNApathwaysDB, RMBase and a series of computational tools for identifying RNA modification sites.

### Help and contact

In the Help module, we provide a simple operation manual to make our RNAWRE convenient to be used. In addition, we also provide information such as our contact addresses, telephone and e-mail in the Contact module to help users to contact us conveniently.

## Discussion and conclusions

Recently, accumulated researches have focused on the recognition of the prevalence and functional significance of RNA modification effectors. Although a huge amount of RNA modification effectors have been identified, a thorough understanding of their functions is still lacking. In order to fill such a knowledge gap, in this study, a database, called RNAWRE, depositing ‘writers’, ‘readers’ and ‘erasers’ of RNA modification was constructed.

Since RNA modification effectors for other modifications are less well described, the current RNAWRE database only contains the effectors for the six major kinds of RNA modifications, namely Cap, m^1^A, m^6^A, m^5^C, ψ and Poly A. Moreover, besides collecting new effectors reported in literatures, we will also collect the effectors from fungi and plants and integrate them into RNAWRE.

Taken together, the RNAWRE database will be helpful for deciphering how these effectors are regulated and integrated in different cell types or tissues. We hope that RNAWRE will provide valuable insights into researches on RNA modifications.

## Funding

National Nature Scientific Foundation of China (31771471); Natural Science Foundation for Distinguished Young Scholar of Hebei Province (C2017209244).
